# Supplementary figures and images for: TOR regulates variability of protein synthesis rates
Source: EMBO J. 2024 Mar 18;43(8):1618–33. doi: 10.1038/s44318-024-00075-8 (PMC11021518; doi:10.1038/s44318-024-00075-8)

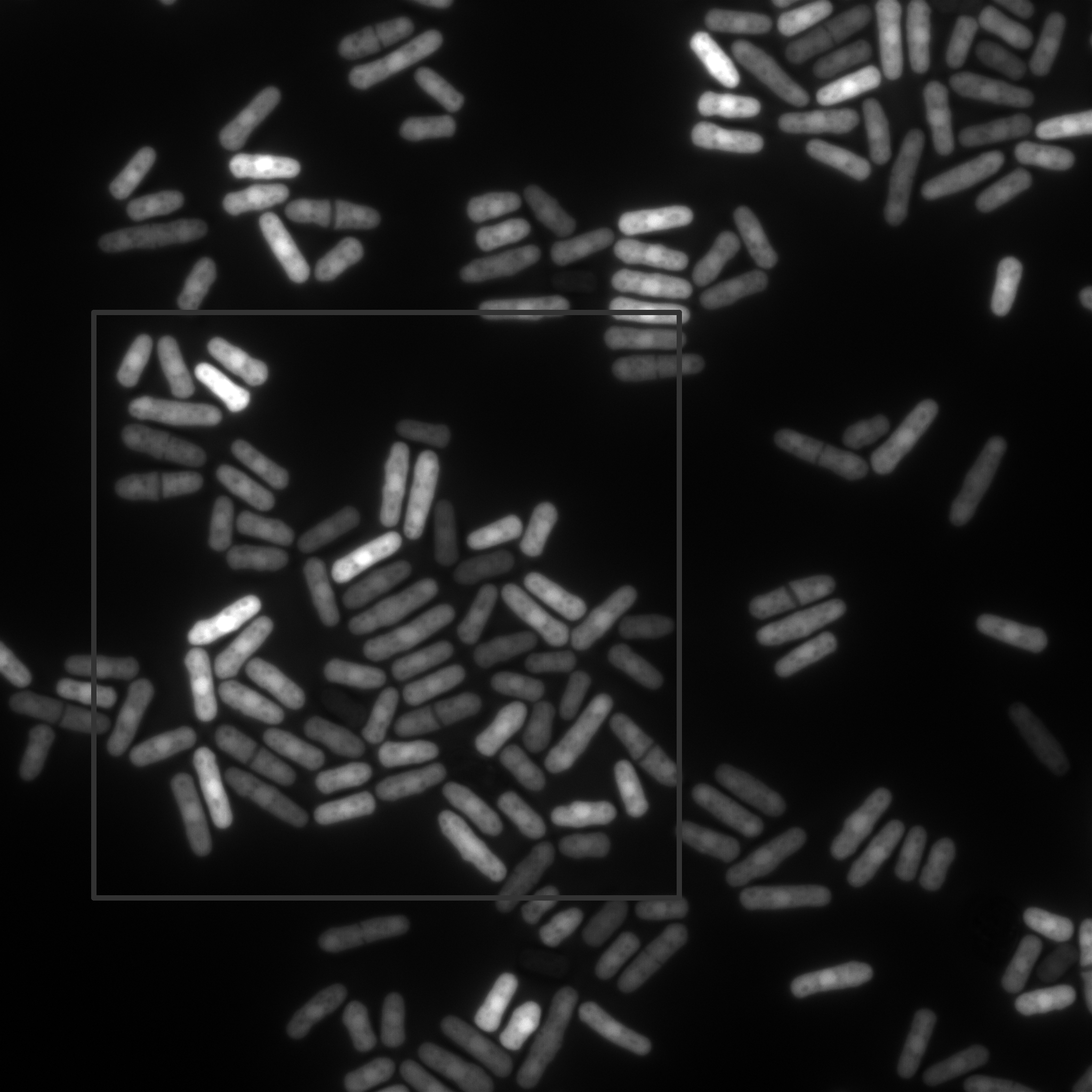

Supplement: Supplementary file 2 — Source Data Fig. 1 [file 44318_2024_75_MOESM2_ESM.zip › Figure 1/C/pn1-1.tif]

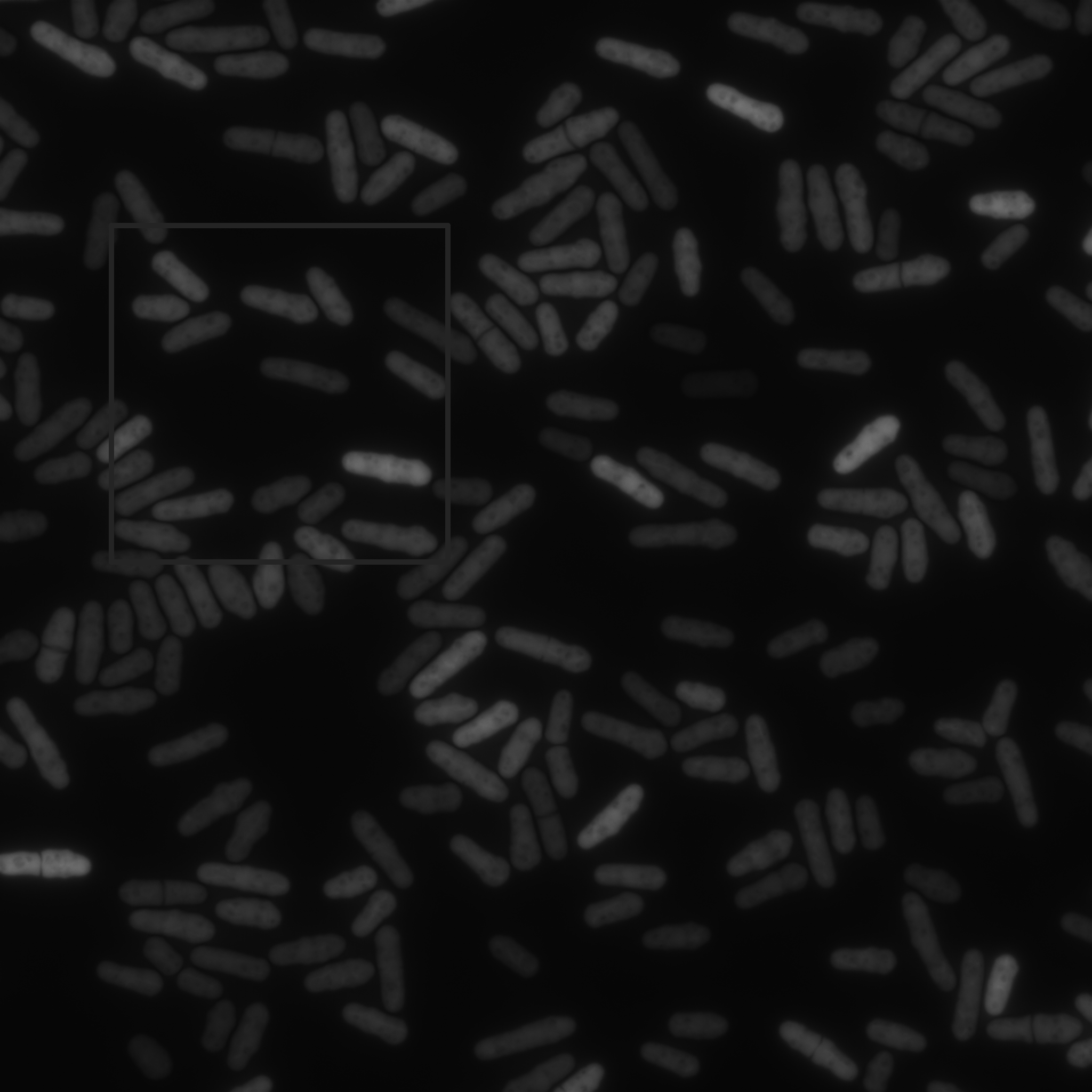

Supplement: Supplementary file 3 — Source Data Fig. 2 [file 44318_2024_75_MOESM3_ESM.zip › Figure 2/C/bottom_two_panels-1.tif]

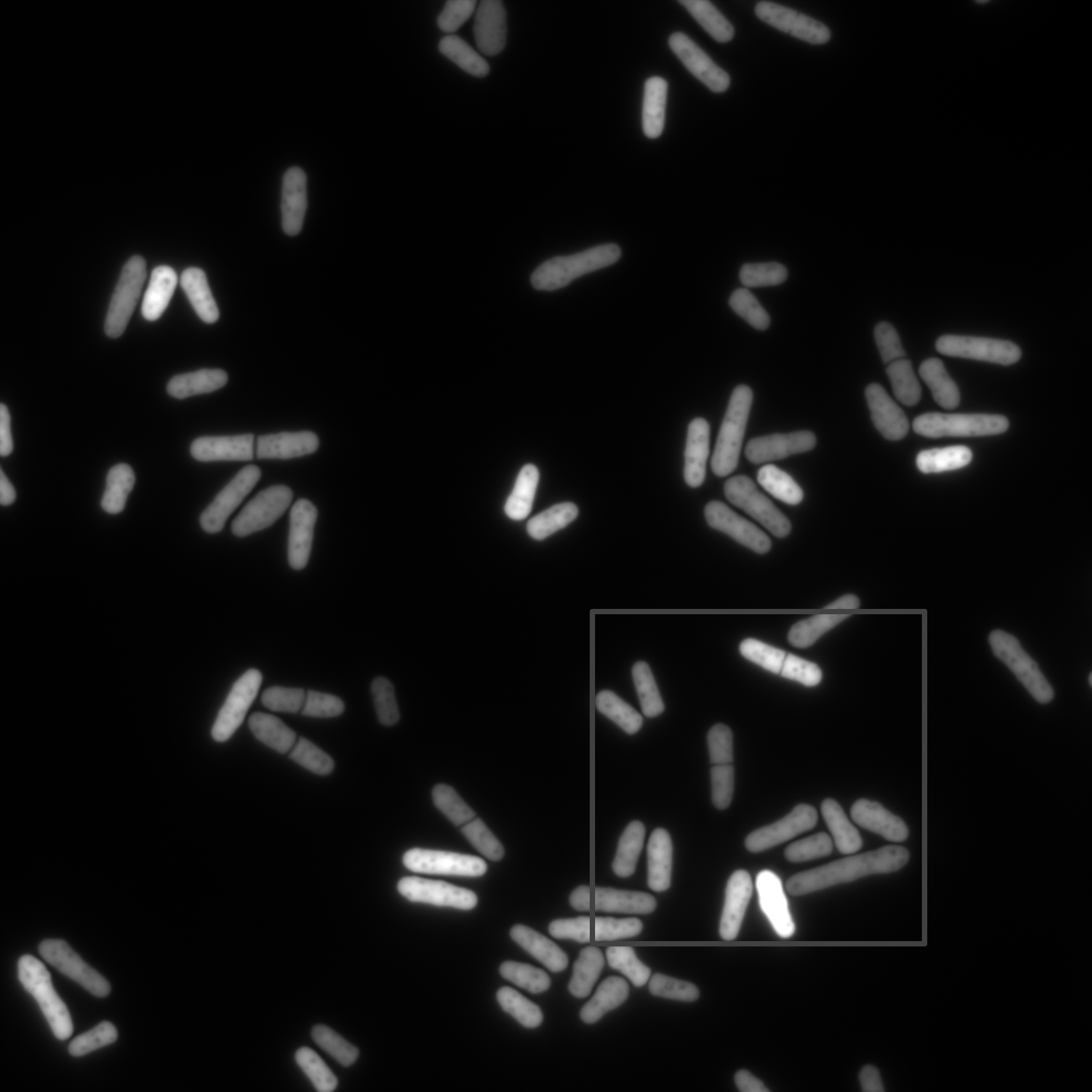

Supplement: Supplementary file 3 — Source Data Fig. 2 [file 44318_2024_75_MOESM3_ESM.zip › Figure 2/C/top_two_panels-1.tif]

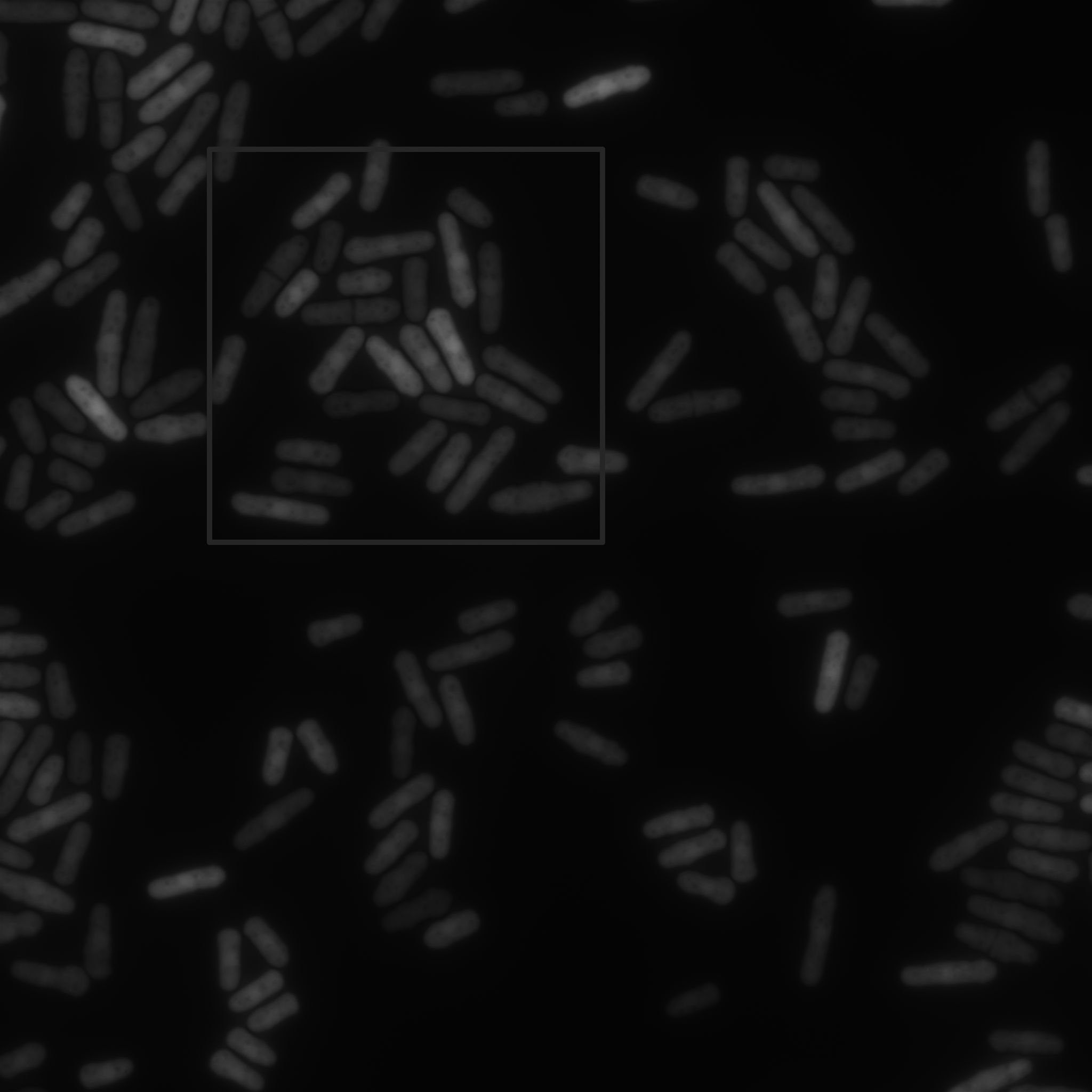

Supplement: Supplementary file 5 — Source Data Fig. 4 [file 44318_2024_75_MOESM5_ESM.zip › Figure 4/A/left_panel-1.tif]

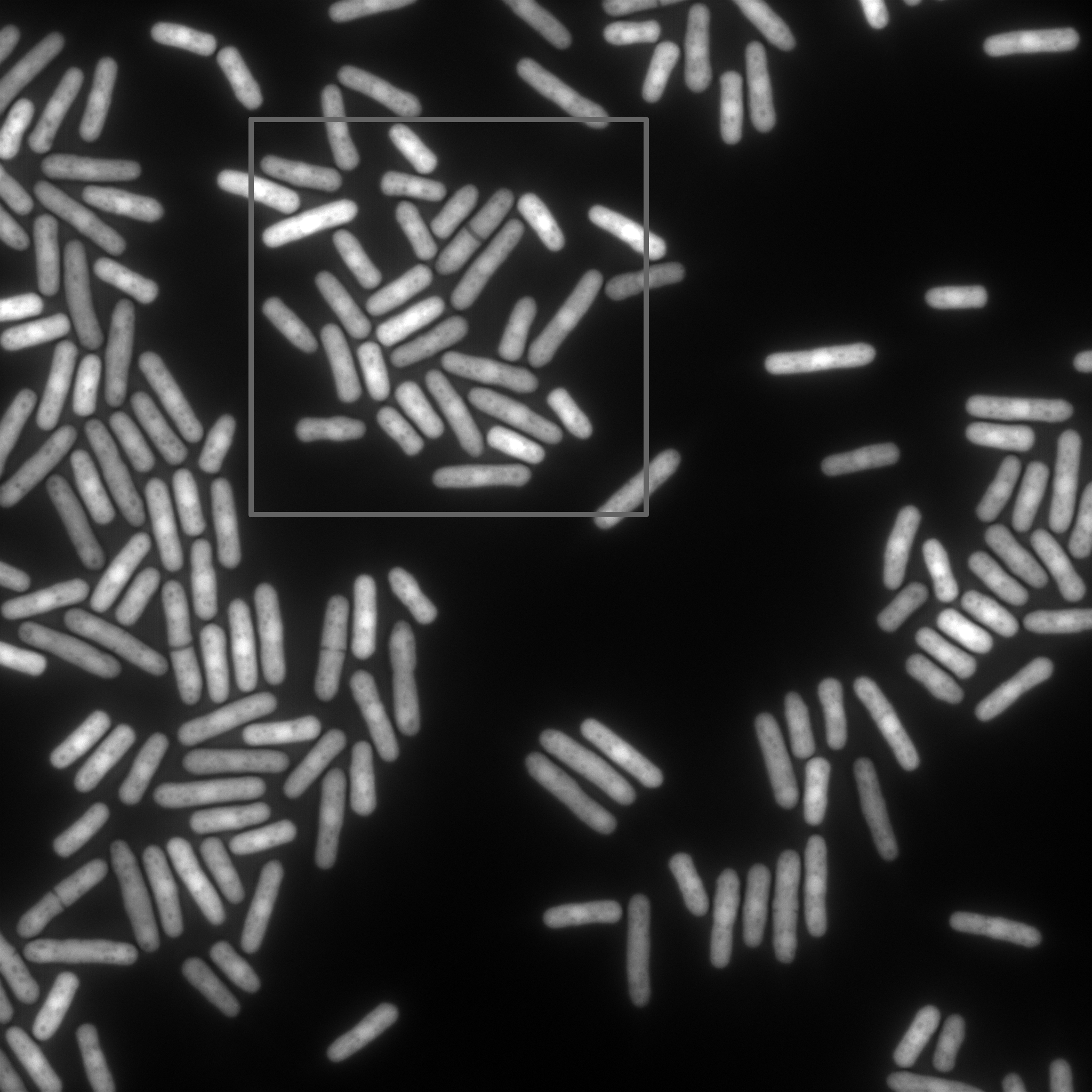

Supplement: Supplementary file 5 — Source Data Fig. 4 [file 44318_2024_75_MOESM5_ESM.zip › Figure 4/A/middle_and_right_panel-1.tif]

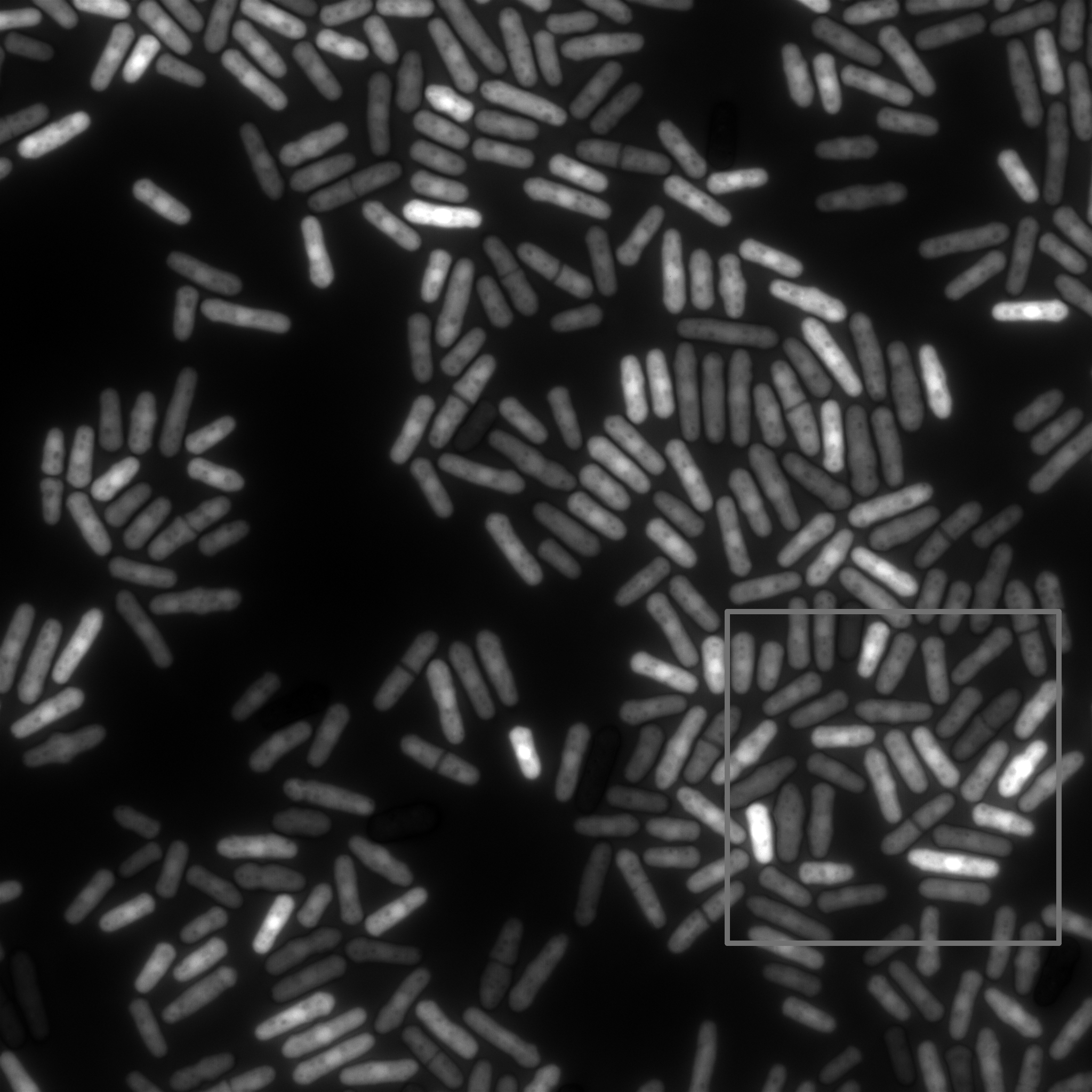

Supplement: Supplementary file 5 — Source Data Fig. 4 [file 44318_2024_75_MOESM5_ESM.zip › Figure 4/B/left_panel-1.tif]

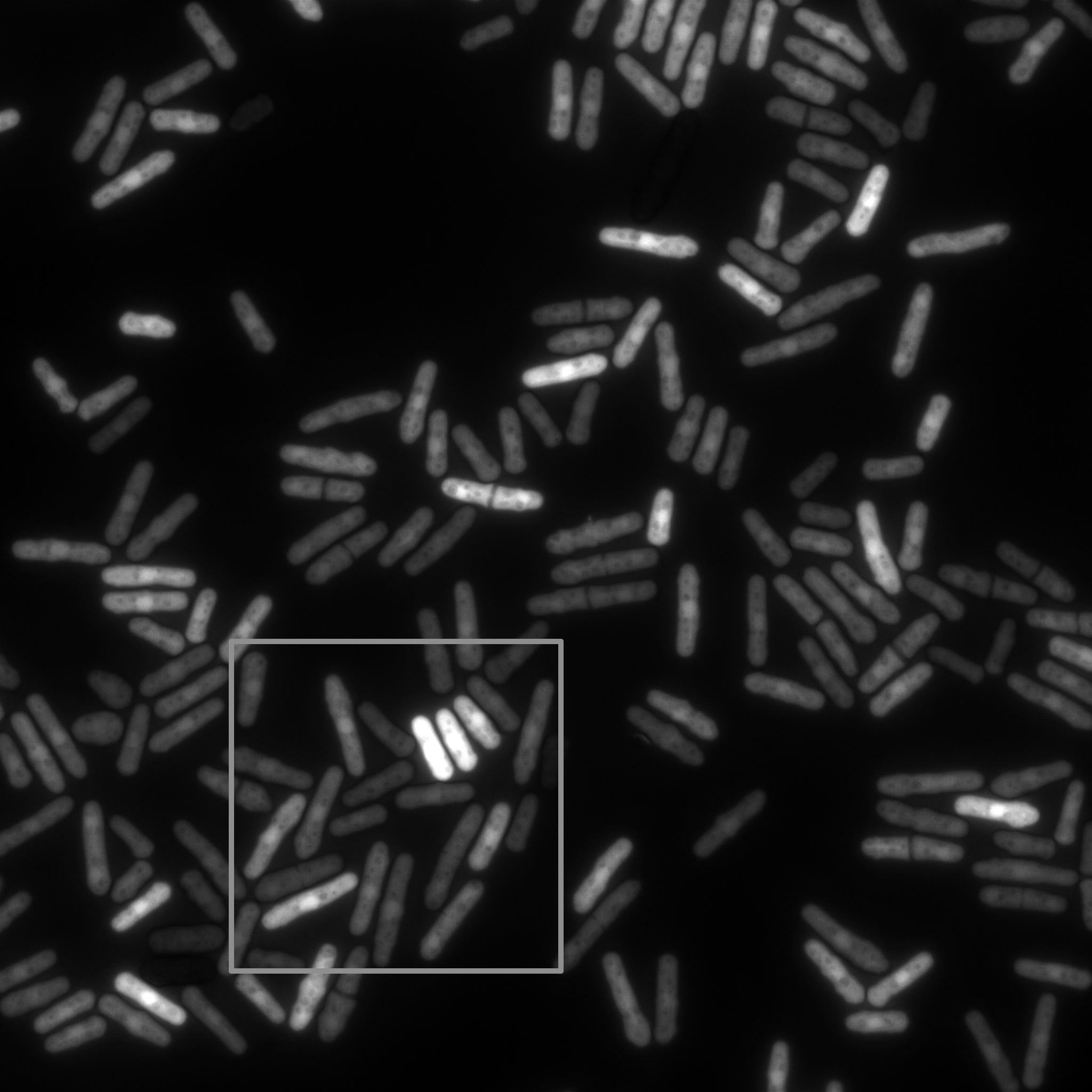

Supplement: Supplementary file 5 — Source Data Fig. 4 [file 44318_2024_75_MOESM5_ESM.zip › Figure 4/B/middle_panel-1.tif]

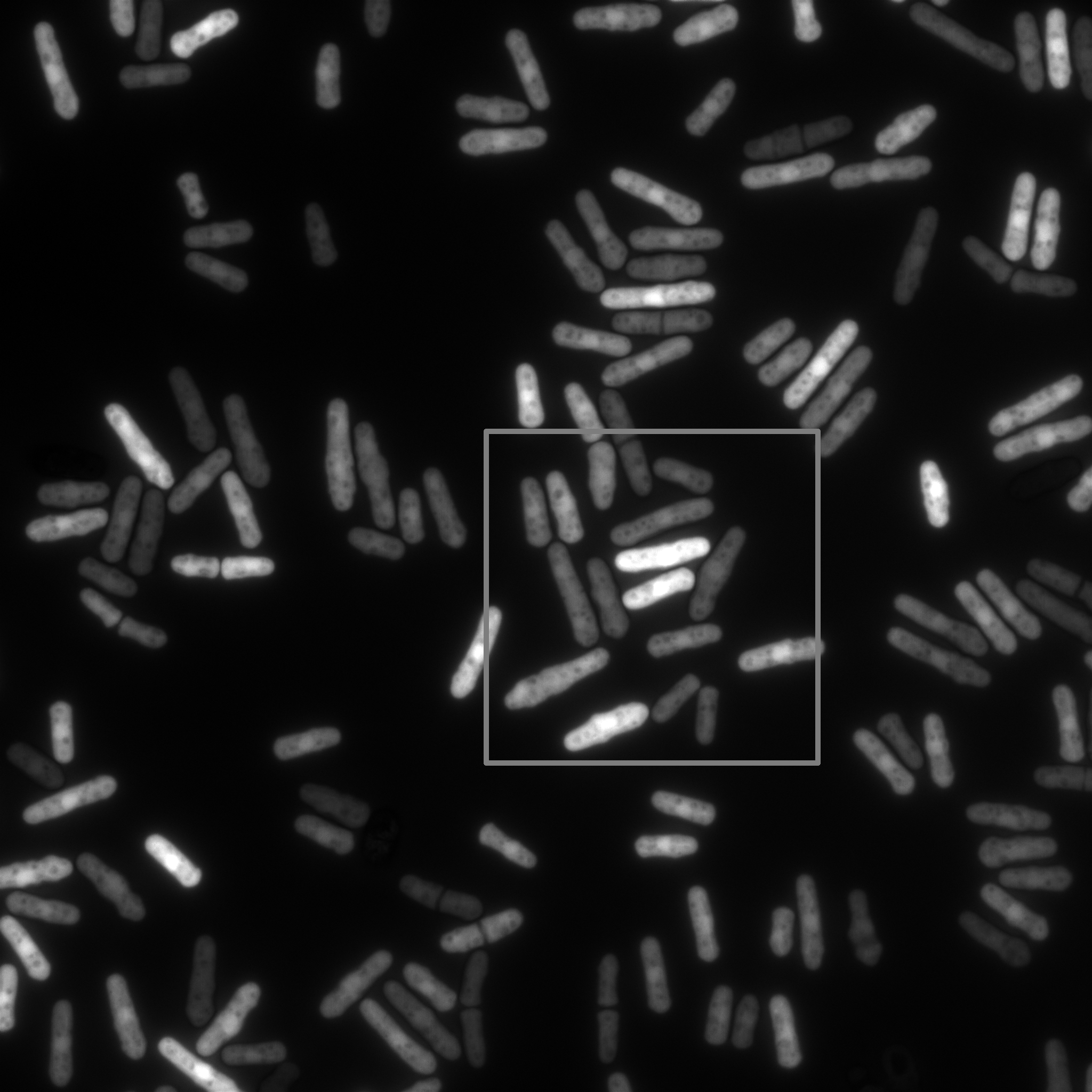

Supplement: Supplementary file 5 — Source Data Fig. 4 [file 44318_2024_75_MOESM5_ESM.zip › Figure 4/B/right_panel-1.tif]
