# Supplementary material for: TOR regulates variability of protein synthesis rates
Source: EMBO J. 2024 Mar 18;43(8):1618–33. doi: 10.1038/s44318-024-00075-8 (PMC11021518; doi:10.1038/s44318-024-00075-8)
Supplement: Supplementary file 3 — Source Data Fig. 2 [file 44318_2024_75_MOESM3_ESM.zip › Figure 2/BDFG/README.rtf]

The single-cell HPG signal with background subtracted (HPG.signal.minus.background) was median normalised (HPG.signal.minus.background.median.norm) and corrected for cell size by normalising by cell area (HPG.signal.minus.background.median.norm.area.norm). The same procedure was applied for the AHA signal. Cells were then split in 10 groups based on their HPG signal rank (group). The HPG.signal.minus.background.median.norm.area.norm and the AHA.signal.minus.background.median.norm.area.norm were used to compute the figures.
