# Supplementary material for: TOR regulates variability of protein synthesis rates
Source: EMBO J. 2024 Mar 18;43(8):1618–33. doi: 10.1038/s44318-024-00075-8 (PMC11021518; doi:10.1038/s44318-024-00075-8)
Supplement: Supplementary file 5 — Source Data Fig. 4 [file 44318_2024_75_MOESM5_ESM.zip › Figure 4/G/README.rtf]

For all the strains assayed on a day, the mean HPG signal per cell (mean.HPG.signal) is normalised by the mean signal of the wild type replicate of the day (mean.HPG.signal.wt.mean.norm) to make them comparable. The distribution of mean.HPG.signal.wt.mean.norm for each strain is used to compute the QCD.
