# Supplementary material for: TOR regulates variability of protein synthesis rates
Source: EMBO J. 2024 Mar 18;43(8):1618–33. doi: 10.1038/s44318-024-00075-8 (PMC11021518; doi:10.1038/s44318-024-00075-8)
Supplement: Supplementary file 5 — Source Data Fig. 4 [file 44318_2024_75_MOESM5_ESM.zip › Figure 4/CE/README.rtf]

Panel C shows data (signal.minus.background.norm) of replicate 3.
