# Supplementary material for: TOR regulates variability of protein synthesis rates
Source: EMBO J. 2024 Mar 18;43(8):1618–33. doi: 10.1038/s44318-024-00075-8 (PMC11021518; doi:10.1038/s44318-024-00075-8)
Supplement: Supplementary file 7 — Expanded View Figures [file 44318_2024_75_MOESM7_ESM.pdf]

Expanded View Figures

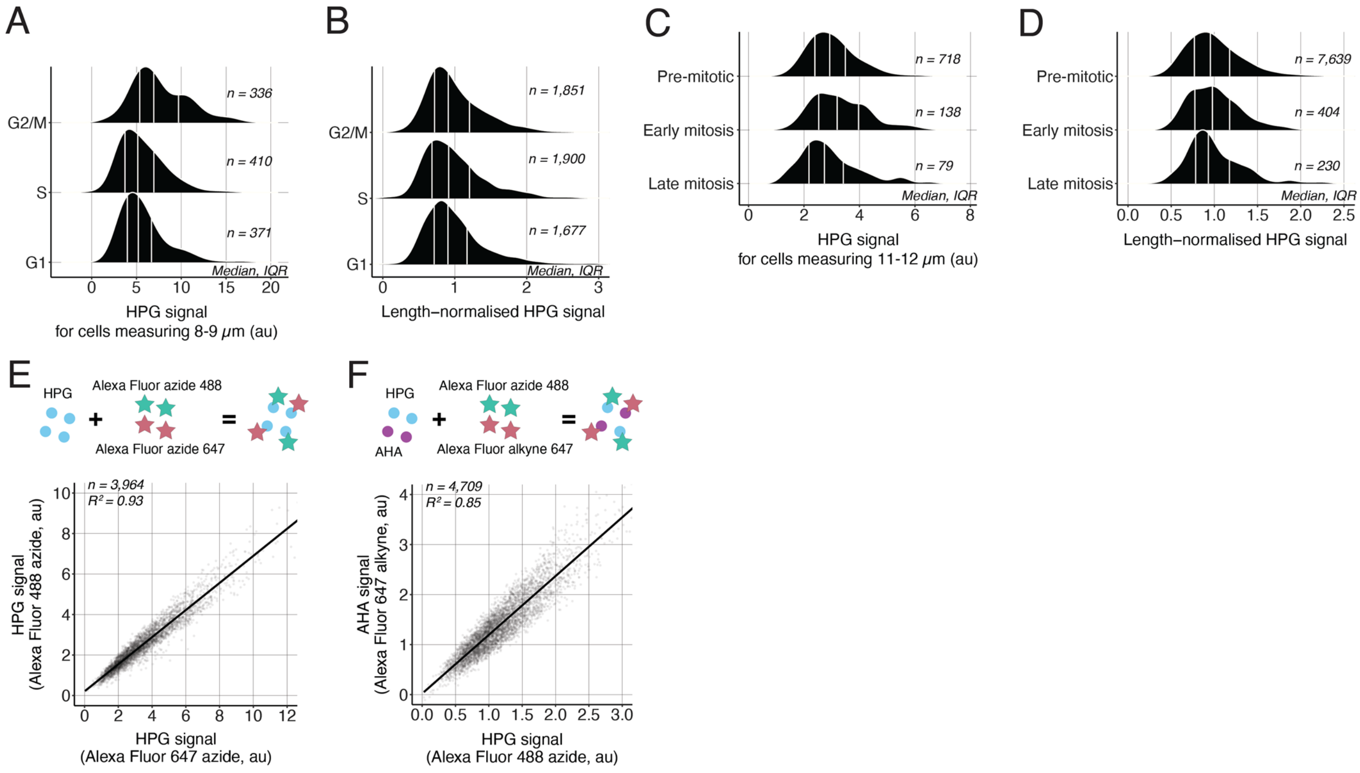

Figure EV1. Influence of the cell cycle stage and the staining procedure on the distribution of the HPG signal.

(**A**) Previously published data of an asynchronous population of *cig1Δ cig2Δ puc1Δ EGFP-pcn1* cells (PN6001) assayed with HPG (Basier and Nurse, 2023) was used to assess the influence of the G1, S, and G2/M cell cycle stages on the distribution of HPG signals. Kernel density estimates of the HPG signal of cells with lengths between 8 and 9 µm. The white lines show the first, second, and third quartiles of the population. (**B**) Kernel density estimates of the length-normalised HPG signal (using the transformation described in Fig. EV3E) for all the *cig1Δ cig2Δ puc1Δ EGFP-pcn1* cells (PN6001) identified as G1, S, or G2/M in the previously published data (Basier and Nurse, 2023). The white lines show the first, second, and third quartiles of the population. (**C**) Previously published data of an asynchronous population of *synCut3-mCherry* cells (PN6004) assayed with HPG (Basier and Nurse, 2023) was used to assess the influence of mitosis on the distribution of HPG signals. Kernel density estimates of the HPG signal of cells with lengths between 11 and 12 µm. The white lines show the first, second, and third quartiles of the population. Pre-mitotic, early mitosis, and late mitosis classifications correspond to "uninucleate, low nuclear synCut3", "uninucleate, high nuclear synCut3", and "binucleate, high nuclear synCut3" in the previously published data, respectively. (**D**) Same as (**B**) for all the *synCut3-mCherry* cells (PN6004) classified in the previously published data (Basier and Nurse, 2023). (**E**) Single-cell measurements of cells incubated for 5 min with 10 µm HPG and co-stained with 2.5 µM Alexa Fluor azide 488 and 2.5 µM Alexa Fluor azide 647 (black dots). The black line is the OLS linear regression fitted on the single-cell data. (**F**) Single-cell measurements of cells co-incubated with 10 µM HPG and 20 µM AHA, and stained with Alexa Fluor azide 488 and Alexa Fluor alkyne 647 (black dots). The black line is the OLS linear regression fitted on the single-cell data.

© The Author(s)

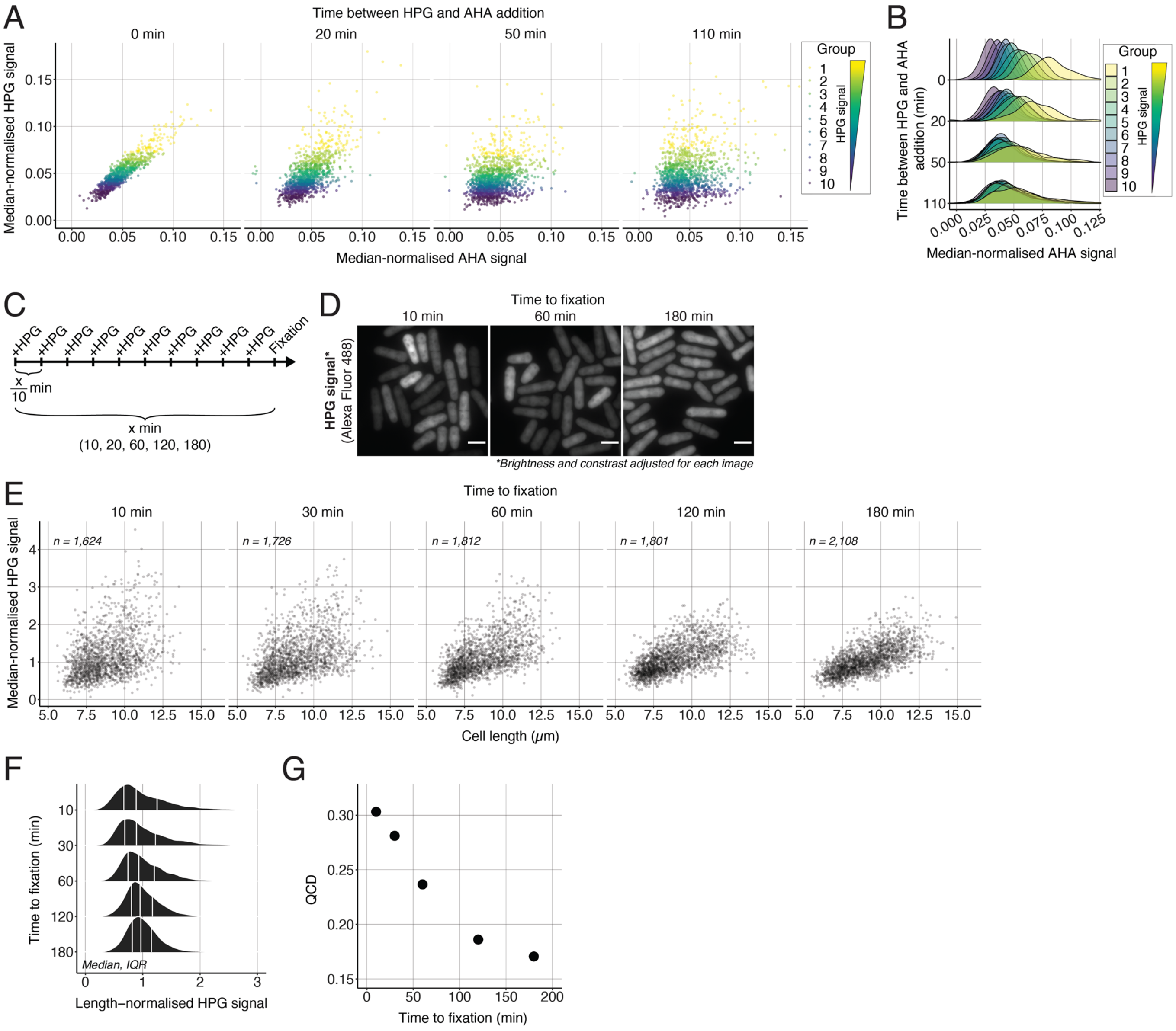

Figure EV2. Dynamics of single-cell variability in the measured global protein synthesis rate.

(**A**) The single-cell data shown in Fig. 2B is colour-coded according to the group attributed to each cell in Fig. 2E–G. (**B**) Same as Fig. 2F for all ten groups. (**C**) Schematic of the repetitive HPG pulses experiment. Wild-type (PN1) cells were incubated with HPG for 10, 30, 60, 120, or 180 min. Each incubation was divided in ten intervals of the same length and 1 μM HPG was added at the beginning of each interval. (**D**) Example images of HPG signal for the 10-, 60-, and 180-minute incubations. The scale bars represent 5 μm. (**E**) For each incubation shown in (**A**), the single-cell HPG signal is divided by the median signal of the incubation. (**F**) Kernel density estimate of the length-normalised HPG signal (using the transformation described in Fig. EV3E) of the populations shown in (**E**). The white lines show the first, second, and third quartiles of the population. (**G**) QCDs of the length-normalised HPG signal distributions shown in (**F**).

 © The Author(s)

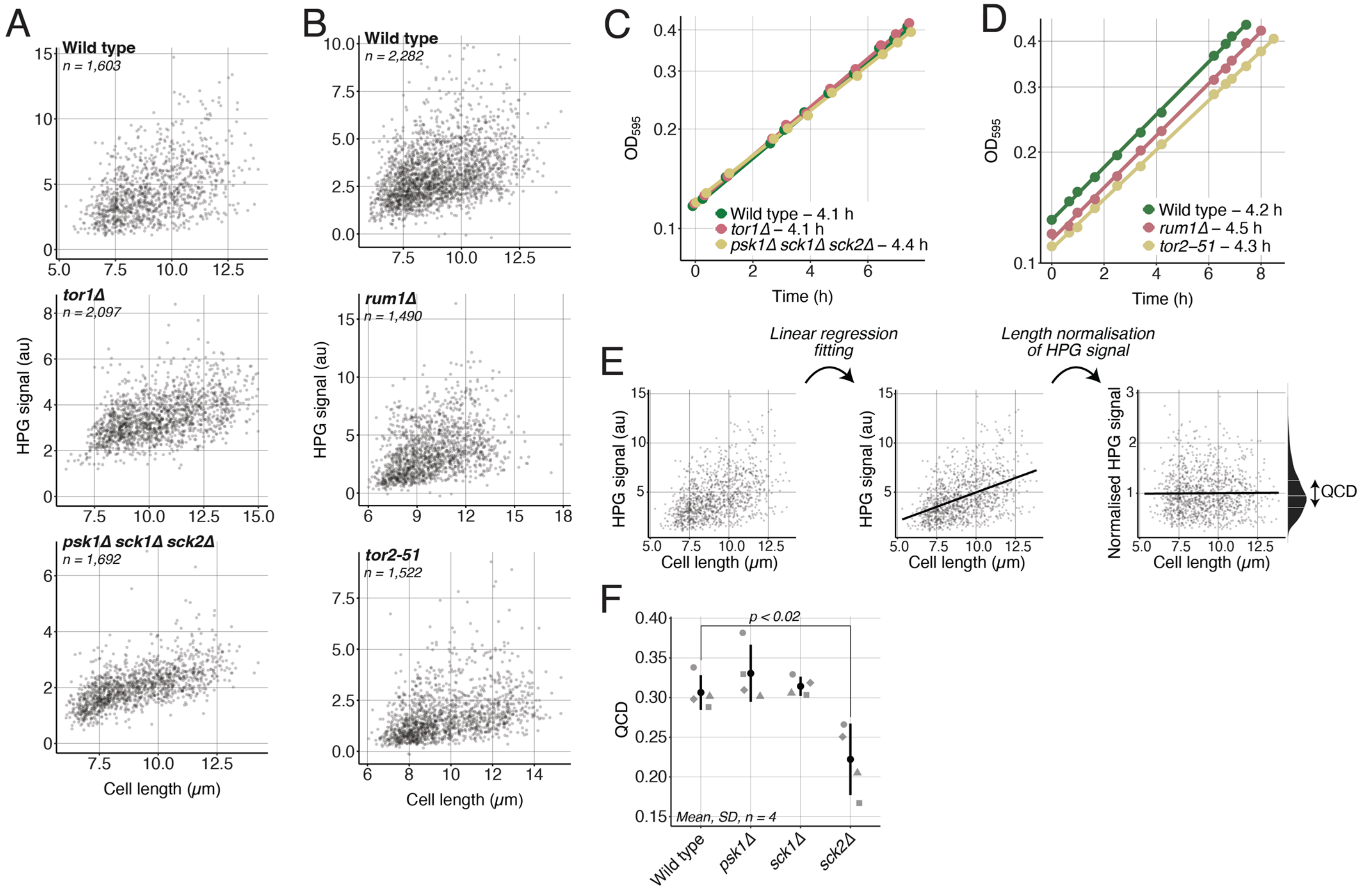

Figure EV3. The TOR pathway and cell cycle controllers modulate variability.

(**A**) Single-cell HPG signal in exponentially growing wild-type fission yeast (PN1), *tor1*Δ (PN5732), and *psk1*Δ *sck1*Δ *sck2*Δ (PN5733) populations of cells. (**B**) Same as (**A**) for wild type (PN1), *rum1*Δ (PN957), and *tor2-51* (PN5413) populations. (**C**) Growth curves of the different strains shown in (**A**). The doubling time of each strain indicated in the legend is calculated using the slope the OLS linear regression fitted on the data after a natural logarithmic transformation. OD_{595} stands for optical density at 595 nm. (**D**) Same as (**C**) for the strains shown in (**B**). (**E**) Schematic of the data processing method used to compute the population QCD after removing the contribution of cell length to the measured variability. An OLS linear regression is fitted on the HPG signal as a function of cell length (black line on the middle panel), then for each cell the normalised HPG signal is obtained by dividing the observed HPG signal by the expected HPG signal for that cell length computed using the OLS linear regression. The distribution to the right of the right panel is the kernel density estimate of the single-cell normalised HPG signal used to compute the QCD. The white lines show the first, second, and third quartiles of the population. (**F**) Mean and standard deviation of the population QCD of four experimental replicates wild type (PN1), *psk1*Δ (PN6084), *sck1*Δ (PN6085), and *sck2*Δ (PN6086). The p values are calculated using a two-sided Welch's unequal variances paired *t* test.

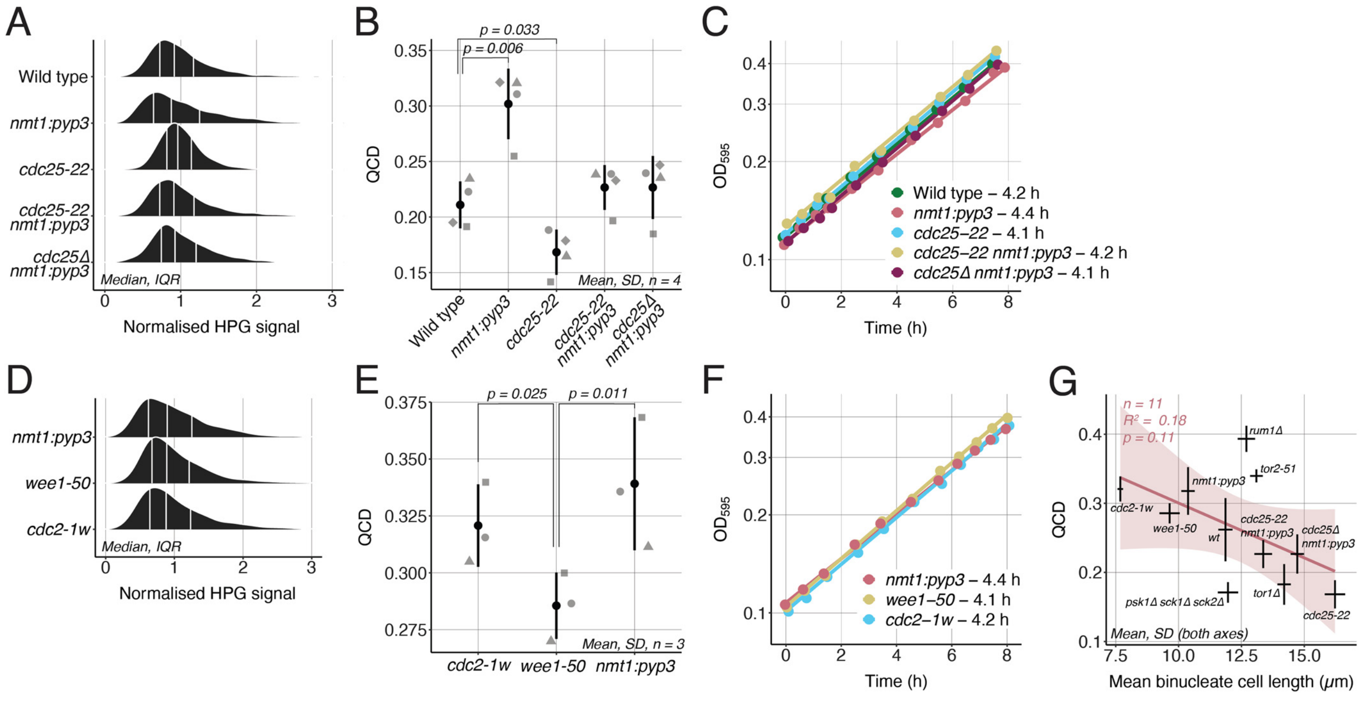

Figure EV4. Cell cycle controllers influence variability.

(**A**) Kernel density estimates of the normalised HPG signal of wild type (PN1), *nmt1:pyp3* (PN6061), *cdc25-22* (PN143), *cdc25-22 nmt1:pyp3* (PN6062), and *cdc25Δ nmt1:pyp3* (PN6060) populations, calculated using the method shown in Figure EV3E. The white lines show the first, second, and third quartiles of the population. (**B**) Mean and standard deviation of the population QCD of four experimental replicates for the different strains shown in (**A**). The *P* values are calculated using a two-sided Welch's unequal variances paired *t* test. (**C**) Growth curves of the different strains shown in (**A**, **B**). The doubling time of each strain indicated in the legend is calculated using the slope the OLS linear regression fitted on the data after a natural logarithmic transformation. OD_{595} stands for optical density at 595 nm. (**D**) Same as (**A**) for *cdc2-1w* (PN150), *wee1-50* (PN369), and *nmt1:pyp3* (PN6061) populations. (**E**) Same as (**B**) for the strains shown in (**D**). (**F**) Same as (**C**) for the strains shown in (**D**, **E**). (**G**) QCD as a function of the mean binucleate cell length in the population for the experimental replicates shown in (**B**), (**F**), and Fig. 4E, F. Mean and standard deviation shown for both axes. For strains appearing on multiple panels, all the experimental replicates are pooled together. The red line represents the OLS linear regression fitted on the mean values for each strain, the shaded area indicates the 95% confidence interval.

 © The Author(s)
